# Comparing the natural progression and clinical features of keratoconus between pediatric and adult patients

**DOI:** 10.1038/s41598-022-12070-2

**Published:** 2022-05-18

**Authors:** Ken-Kuo Lin, Yun-Wen Chen, Chun-Ting Yeh, Pei-Ru Li, Jiahn-Shing Lee, Chiun-Ho Hou, Ching-Hsi Hsiao, Lai-Chu See

**Affiliations:** 1 Department of Ophthalmology, Chang Gung Memorial Hospital at Linkou, Taoyuan City, Taiwan; 2 School of Medicine, College of Medicine, Chang Gung University, Taoyuan City, Taiwan; 3 Department of Ophthalmology, Chang Gung Memorial Hospital at Kaohsiung, Kaohsiung City, Taiwan; 4 Department of Public Health, College of Medicine, Chang Gung University, 259, Wenhua 1st Rd., Guishan Dist., Taoyuan City, 33302 Taiwan; 5 Molecular Medicine Research Center, Chang Gung University, Taoyuan City, Taiwan; 6 Division of Rheumatology, Allergy and Immunology, Chang Gung Memorial Hospital at Linkou, Taoyuan City, Taiwan

**Keywords:** Eye manifestations, Corneal diseases

## Abstract

To present the natural course of keratoconus (KC) and compare pediatric and adult patients. *Design* A retrospective cohort study. *Setting* Hospital-based. *Patient Population* In total, 152 patients (288 eyes) diagnosed with KC at Chang Gung Memorial Hospital, Taiwan, were included. Previously managed patients and those with missing optical data were excluded. *Observation Procedures* Patients were divided into pediatric (≤ 18 years) and adult (> 18 years) groups. Demographics, clinical data, and optical variables were collected, including corrected distance visual acuity (CDVA), refractive error, and keratometric readings (K). *Main Outcome Measure* Optical variables at the final follow-up before aggressive treatment. *Results* In total, 20 pediatric (37 eyes) and 132 adults (251 eyes) patients were eligible for this study. The mean follow-up time was 2.98 years. Male predominance was observed in both groups. Both groups had similar clinical characteristics and optical variables at the initial diagnosis. Pediatric patients progressed significantly more rapidly in refractive errors, including spheres and cylinders, spherical equivalence, steep K, and flat K during the follow-up. However, significant change between the two study groups was only seen in sphere refractive error spherical equivalence. *Conclusion* Pediatric patients had more rapidly progressive KC than adult patients, so early detection and frequent follow-up for prompt interventions are necessary for these patients.

## Introduction

Keratoconus (KC), although traditionally classified as a rare disease, has an estimated prevalence of 1 in 375^1^ to 2000^2^ and is a crucial contributor to visual debilitation in the young population^3–5^. It has been reported that the prevalence of KC was higher in Asian countries^6^. More importantly, progressive ectasia and bilateral and asymmetrical corneal distortion ultimately affect these patients’ vision-related quality of life if the patients do not receive appropriate management^7,8^.

With the advancement of corneal imaging technology, early detection and interventions are becoming feasible. However, the treatment method is mainly determined by the pattern of disease progression and the severity of corneal ectasia^9–11^. Optical correction with spectacles or rigid contact lenses could effectively enhance the quality of vision in patients with slow disease progression. In comparison, corneal cross-linking (CXL) has shown promising results in halting rapid disease progression.

A detailed understanding of the natural history of KC is fundamental in making informed decisions on when their benefits outweigh the risks^12^. The natural course refers to the progression of a disease process in an individual over time without aggressive treatment. Any treatment modalities that contact the cornea, including contact lenses or surgery, are considered an aggressive treatment for KC. In other words, correcting the visual acuity with spectacles is not an aggressive treatment for KC. Hence, the disease process from the initial diagnosis of KC up to treating with contact lenses or surgery is the natural course of KC.

Unfortunately, the natural history of KC is poorly understood^12–14^. Ferdi et al. included 41 studies in their systematic review and 23 studies into their meta-analysis to describe the natural progression of KC based on 11,529 untreated eyes. They reported that younger patients (17 years old or less) and those with Kmax steeper than 55 D at presentation have a significantly greater risk of progression of the disease^12^. However, this systemic review and meta-analysis analyzed data at 12 months due to inconsistent follow-up duration across various studies, and most patients with KC were adults. Compared with adults, KC in children progresses more rapidly and is usually more severe at the time of diagnosis^15^. In addition, age may influence corneal cross-linking treatment in KC. A study of 119 KC eyes reported that pediatric cornea showed a more flattening effect after cross-linking than older patients and the better-corrected distance visual acuity after cross-linking^16^. Therefore, there is a need to identify the differences in disease progression between pediatric and adult patients with KC and explore the overall risk stratification of natural disease progression.

There were multiple parameters for monitoring KC progressions, such as CDVA^17^, maximum keratometry^17^, Scheimpflug from Pentacam^18,19^, or swept-source optical coherence tomography^20^. Amsler-Krumeich classification proposed in 1947 was used to grade KC based on keratometry and optical pachymetry^21,22^. The Belin ABCD classification introduced on the Pentacam has advantages in presenting anatomical changes seen in KC and other ectatic diseases^22^. In 2019, Robert et al. represented the Dutch Crosslinking for Keratoconus (DUCK) based on five domains: age, decrease in visual acuity, increase in refractive error, increase in keratometry readings, and perceived quality of vision for determining whether a cross-linking might be warranted^23^.

In this study, we did a chart review of patients with KC in Chang Gung Medical Hospital (CGMH), Taiwan, and aimed to compare the KC’s clinical characteristics and natural progression among two age groups in Taiwan.

## Results

A total of 288 eyes of 152 patients were analyzed. We divided the patients into two groups depending on age, ≤ 18 years (*n* = 20) and > 18 years (*n* = 132). Male predominance was observed in both pediatric (65.0%) and adult (62.1%) groups. In the pediatric and adult groups, the mean age of KC diagnosis was 15.3 years and 27.4 years. The prevalence of atopic dermatitis was 5.0% and 6.1%, and the history of eye rubbing was 20.0% and 11.4%, respectively. Although the eye rubbing rate in the pediatric group was higher than the adult group, there was no significant difference between the two study groups (*p* = 0.2810). In 288 eyes, 32 (11.1%) presented with Vogt’s striae, and 25 eyes (8.7%) had Fleischer ring. The cones were recorded as nipple (11.5%), oval (55.9%), and globus (32.6%). There were similar results in Vogt’s striae or Fleischer ring, cone morphology, and location between the two study groups. In brief, there were no significant differences between pediatric and adult groups with clinical appearance, comorbidity, cone morphology, and location (Table 1).

**Table 1:** Demographic and clinical characteristics of patients with keratoconus at the initial diagnosis.

	Total	Pediatric (≤ 18 y/o)	Adult (> 18 y/o)	*p*-value
Number of patients	152	20 (13.2%)	132 (86.8%)	
Number of eyes	288	37 (12.8%)	251 (87.2%)	
Laterality				1.0000^a^
OD	146	19 (51.3%)	127 (50.6%)	
OS	142	18 (48.7%)	124 (49.4%)	
Sex				1.0000^a^
Male	95 (62.5%)	13 (65.0%)	82 (62.1%)	
Female	57 (37.5%)	7 (35.0%)	50 (37.9%)	
Age at KC diagnosis (years)				
Median (IQR)	24.2 (11.2)	15.1 (3.4)	24.8 (10.7)	< .0001^b^*
Mean ± SD	25.8 ± 8.4	15.3 ± 1.8	27.4 ± 7.9	
Clinical appearance				
Vogt's striae	32 (11.1%)	3 (8.1%)	29 (11.6%)	0.7794^a^
Fleischer ring	25 (8.7%)	4 (10.8%)	21 (8.4%)	0.5424^a^
Cone morphology				0.7147^a^
Nipple	33 (11.5%)	4 (10.8%)	29 (11.5%)	
Oval	161 (55.9%)	23 (62.2%)	138 (55.0%)	
Globus	94 (32.6%)	10 (27.0%)	84 (33.5%)	
Cone location				0.3582^a^
Central	189 (65.6%)	27 (73.0%)	162 (64.5%)	
Paracentral	99 (34.4%)	10 (27.0%)	89 (35.5%)	
Comorbidity				
Atopic dermatitis	9 (5.9%)	1 (5.0%)	8 (6.1%)	1.0000^a^
Eye rubbing	19 (12.5%)	4 (20.0%)	15 (11.4%)	0.2810^a^

^a^Fisher exact test. ^b^Wilcoxon rank-sum test. KC: keratoconus. OD: right eye. OS: left eye. y/o: years old. SD: standard deviation. *statistical significance according to the sequential Bonferroni-type procedure, because the smallest *p* was < i/46* 0.05 for the 41multiple tests in Tables 1 and 2.

To study the natural progression of KC, we defined the follow-up time as the interval between the initial diagnosis and the final visit of the untreated patients or just before they underwent any interventions. The mean follow-up time was 2.98 ± 3.96 years. At the initial diagnosis, pediatric patients had better CDVA (LogMAR) and sphere refractive error than adult ones (*p* = 0.0546 and *p* = 0.0539, respectively). There were no differences in cylinder refractive error, spherical equivalence, steep K, and flat K (*p* = 0.2055, *p* = 0.1352, *p* = 0.8570, and *p* = 0.2688, respectively). For the entire group, the CDVA, sphere refractive error, cylinder refractive error, and spherical equivalence progressed significantly in the final visit compared with the initial presentation (*p* = 0.0048, *p* = 0.0092, *p* = 0.0037, *p* = 0.0015, respectively), while there was no significant progression in steep K and flat K. In the pediatric group, significant or borderline significant progression was seen in sphere refractive error (*p* = 0.0130), cylinder refractive error (*p* = 0.0238), spherical equivalence (*p* = 0.0052), steep K (*p* = 0.0339) and flat K (*p* = 0.0365). In the adult group, borderline significant progression was seen in CDVA (*p* = 0.0235), cylinder refractive error (*p* = 0.0407). The significance progression between the study groups was seen for sphere refractive error (*p* = 0.0071) and spherical equivalence (*p* = 0.0066), respectively (Table 2).

**Table 2:** Optical variables of patients with keratoconus at the initial diagnosis and the final visit before intervention.

	Total	Pediatric	Adult	p-value
Number of eyes included	288	37 (12.9%)	251 (87.1%)	
**Follow-up duration**^**a**^** (years)**				
Median (IQR)	1.18 (4.24)	3.16 (2.95)	0.75 (4.22)	0.0447^b^
Mean ± SD	2.98 ± 3.96	3.67 ± 3.28	2.88 ± 4.05	
Min–max		1.66–13.65	0.02–15.55	
**CDVA (LogMAR)**				
Initial diagnosis				
Median (IQR)	0.15 (0.40)	0.05 (0.22)	0.15 (0.52)	0.0546^b^
Mean ± SD	0.30 ± 0.41	0.15 ± 0.21	0.32 ± 0.43	
**Final visit**				
Median (IQR)	0.22 (0.52)	0.10 (0.40)	0.22 (0.52)	0.1767^b^
Mean ± SD	0.34 ± 0.40	0.25 ± 0.33	0.35 ± 0.41	
**Difference**				
Median (IQR)	0.00 (0.00)	0.00 (0.15)	0.00 (0.00)	0.5196^b^
Mean ± SD	0.03 ± 0.22	0.10 ± 0.29	0.03 ± 0.20	
*p*-value	0.0048^c^*	0.1126^c^	0.0235^c^	
**Sphere refractive error (diopter)**				
Initial diagnosis				
Median (IQR)	− 5.00 (5.25)	− 3.75 (3.50)	− 5.25 (5.75)	0.0539^b^
Mean ± SD	− 5.84 ± 4.68	− 4.37 ± 3.37	− 6.04 ± 4.80	
**Final visit**				
Median (IQR)	− 5.38 (5.25)	− 4.75 (4.25)	− 5.50 (5.75)	0.8349^b^
Mean ± SD	− 6.16 ± 4.79	− 6.12 ± 4.72	− 6.16 ± 4.81	
**Difference**				
Median (IQR)	0.00 (0.00)	0.00 (2.50)	0.00 (0.00)	0.0071^b^*
Mean ± SD	− 0.32 ± 2.26	− 1.75 ± 4.34	− 0.13 ± 1.74	
*p*-value	0.0092^c^	0.0130^c^	0.1625^c^	
**Cylinder refractive error (diopter)**				
Initial diagnosis				
Median (IQR)	− 3.75 (3.75)	− 4.50 (3.00)	− 3.75 (4.00)	0.2055^b^
Mean ± SD	− 3.88 ± 2.35	− 4.33 ± 2.02	− 3.81 ± 2.39	
**Final visit**				
Median (IQR)	− 4.00 (4.25)	− 5.00 (4.00)	− 3.75 (4.25)	0.0238^b^
Mean ± SD	− 4.10 ± 2.57	− 5.21 ± 2.63	− 3.95 ± 2.53	
**Difference**				
Median (IQR)	0.00 (0.25)	0.00 (1.50)	0.00 (0.25)	0.1396^b^
Mean ± SD	− 0.23 ± 1.34	− 0.88 ± 1.78	− 0.14 ± 1.24	
*p*-value	0.0037^c^*	0.0238^c^	0.0407^c^	
**Spherical equivalence (diopter)**				
Initial diagnosis				
Median (IQR)	− 6.81 (5.13)	− 5.75 (3.75)	− 7.00 (5.13)	0.1352^b^
Mean ± SD	− 7.85 ± 4.88	− 6.54 ± 3.43	− 8.03 ± 5.03	
**Final visit**				
Median (IQR)	− 7.50 (5.88)	− 8.13 (4.00)	− 7.38 (6.25)	0.5219^b^
Mean ± SD	− 8.23 ± 5.01	− 8.73 ± 5.01	− 8.16 ± 5.02	
**Difference**				
Median (IQR)	0.00 (0.13)	0.00 (3.00)	0.00 (0.00)	0.0066^b^*
Mean ± SD	− 0.44 ± 2.39	− 2.19 ± 4.50	− 0.20 ± 1.81	
*p*-value	0.0015^c^*	0.0052^c^*	0.0572^c^	
**Steep K (diopter)**				
Initial diagnosis				
Median (IQR)	48.63 (6.25)	48.75 (6.75)	48.63 (6.25)	0.8570^b^
Mean ± SD	49.57 ± 5.00	49.64 ± 4.85	49.56 ± 5.03	
**Final visit**				
Median (IQR)	48.75 (6.50)	49.00 (8.00)	48.75 (6.00)	0.5463^b^
Mean ± SD	49.73 ± 5.30	50.38 ± 5.47	49.64 ± 5.28	
**Difference**				
Median (IQR)	0.00 (0.00)	0.00 (0.50)	0.00 (0.00)	0.0934^b^
Mean ± SD	0.16 ± 2.04	0.74 ± 1.81	0.08 ± 2.07	
*p*-value	0.1769^c^	0.0339^c^	0.5833^c^	
**Flat K (diopter)**				
Initial diagnosis				
Median (IQR)	44.25 (3.92)	44.00 (4.10)	44.38 (4.00)	0.2688^b^
Mean ± SD	45.27 ± 4.08	44.71 ± 3.60	45.36 ± 4.15	
**Final visit**				
Median (IQR)	44.25 (3.88)	43.75 (5.00)	44.25 (3.75)	0.4998^b^
Mean ± SD	45.42 ± 4.25	45.31 ± 4.15	45.43 ± 4.27	
**Difference**				
Median (IQR)	0.00 (0.00)	0.00 (0.75)	0.00 (0.00)	0.3702^b^
Mean ± SD	0.14 ± 1.45	0.60 ± 1.58	0.08 ± 1.42	
*p*-value	0.1065^c^	0.0365^c^	0.4810^c^	

^a^from the initial diagnosis of KC to the final visit before intervention. ^b^Wilcoxon rank-sum test. ^c^Wilcoxon signed-rank test. K: keratometry readings. CDVA: corrected distance visual acuity. SD: standard deviation.

*Statistical significance according to the sequential Bonferroni-type procedure, because the smallest *p* was < i/46* 0.05 for the 46 multiple tests in Tables 1 and 2, and i is the ranking of the p values of multiple tests from smallest to largest as p(i).

**Figure 1:**
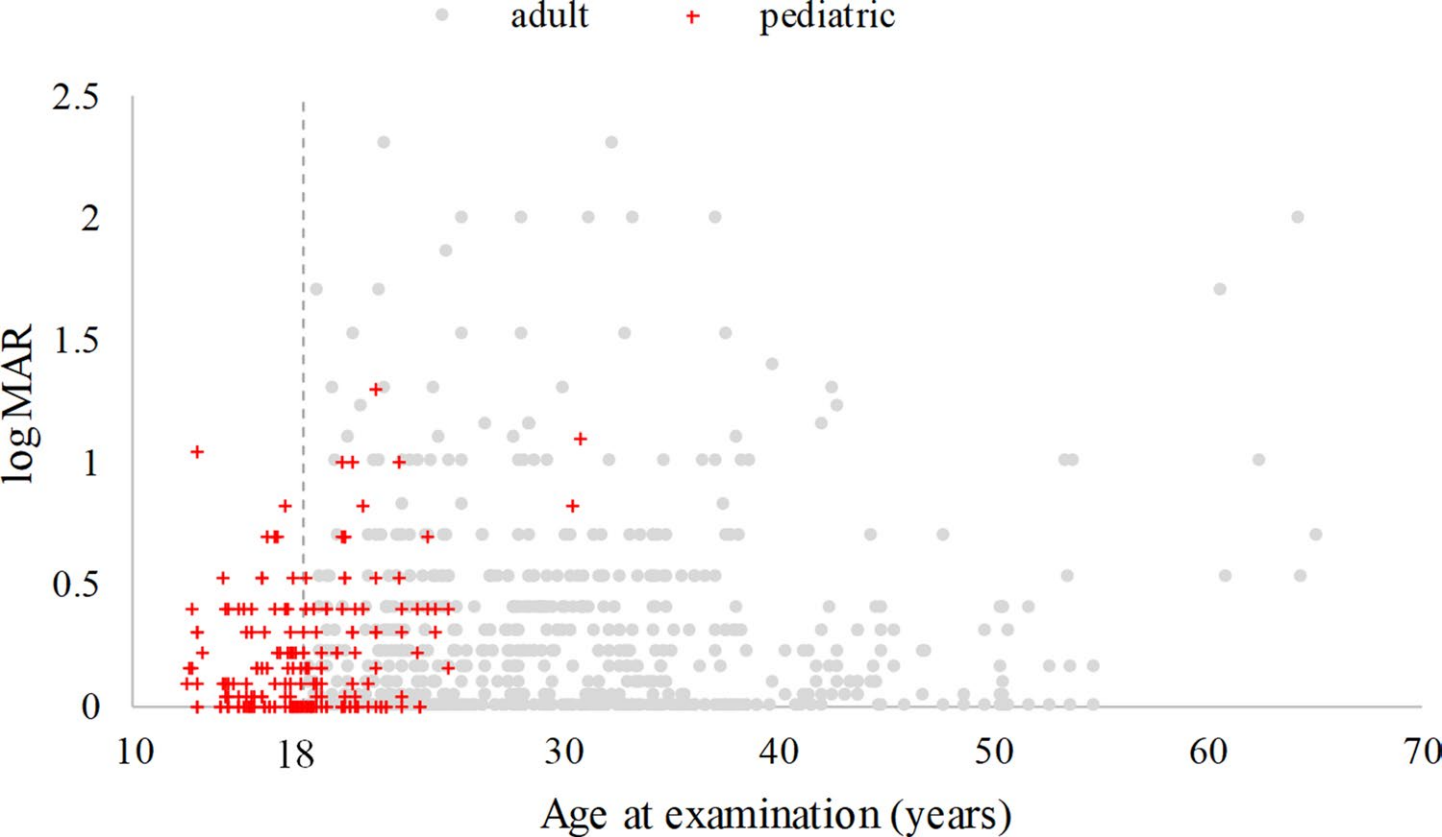
Corrected distance visual acuity (CDVA) in Log MAR and age among patients with keratoconus from the initial diagnosis to the final visit before treatment (867 observations, r = 0.09).

**Figure 2:**
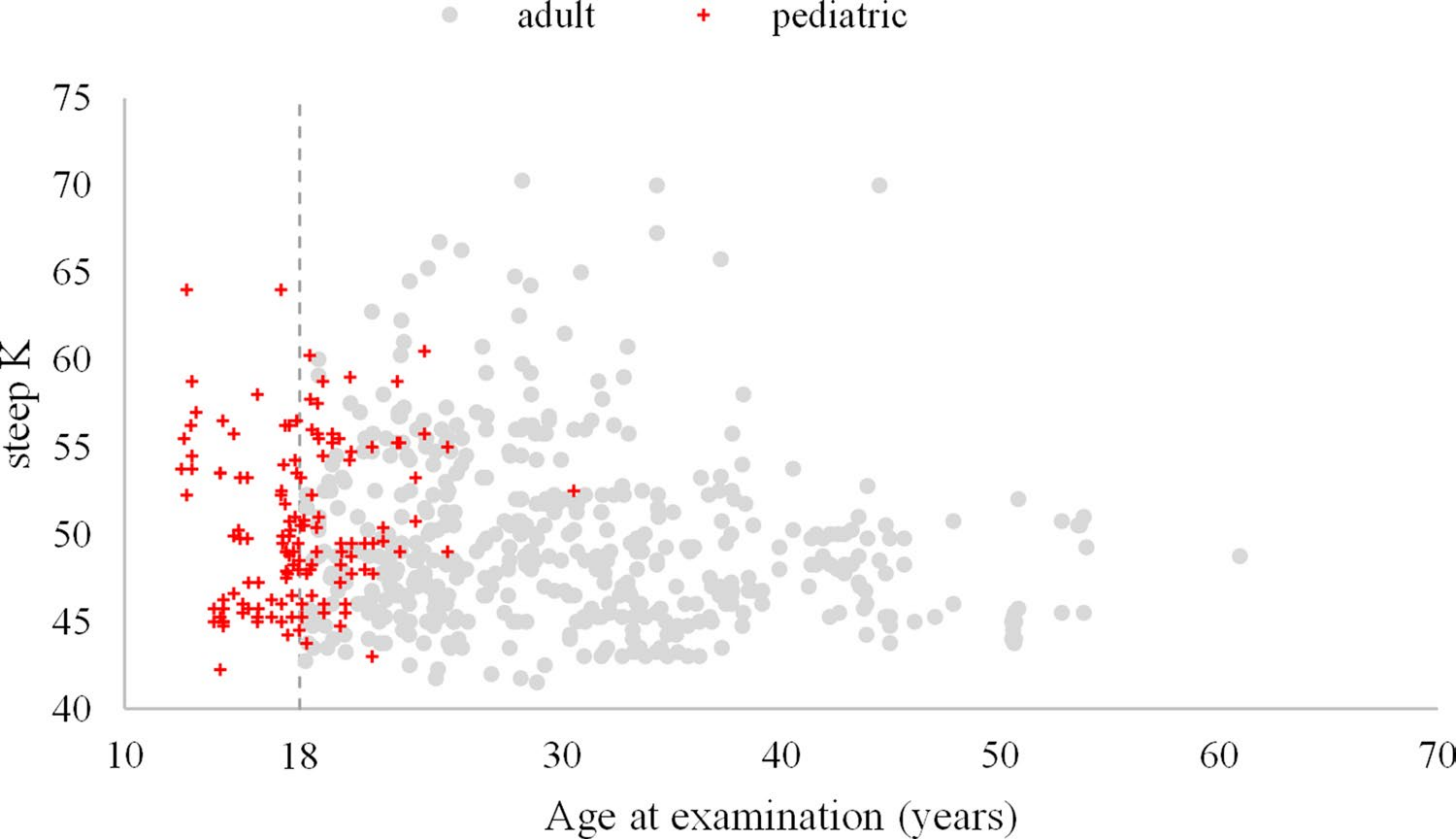
Steep K and age among patients with keratoconus from the initial diagnosis to the final visit before treatment (867 observations, r = 0.14).

**Figure 3:**
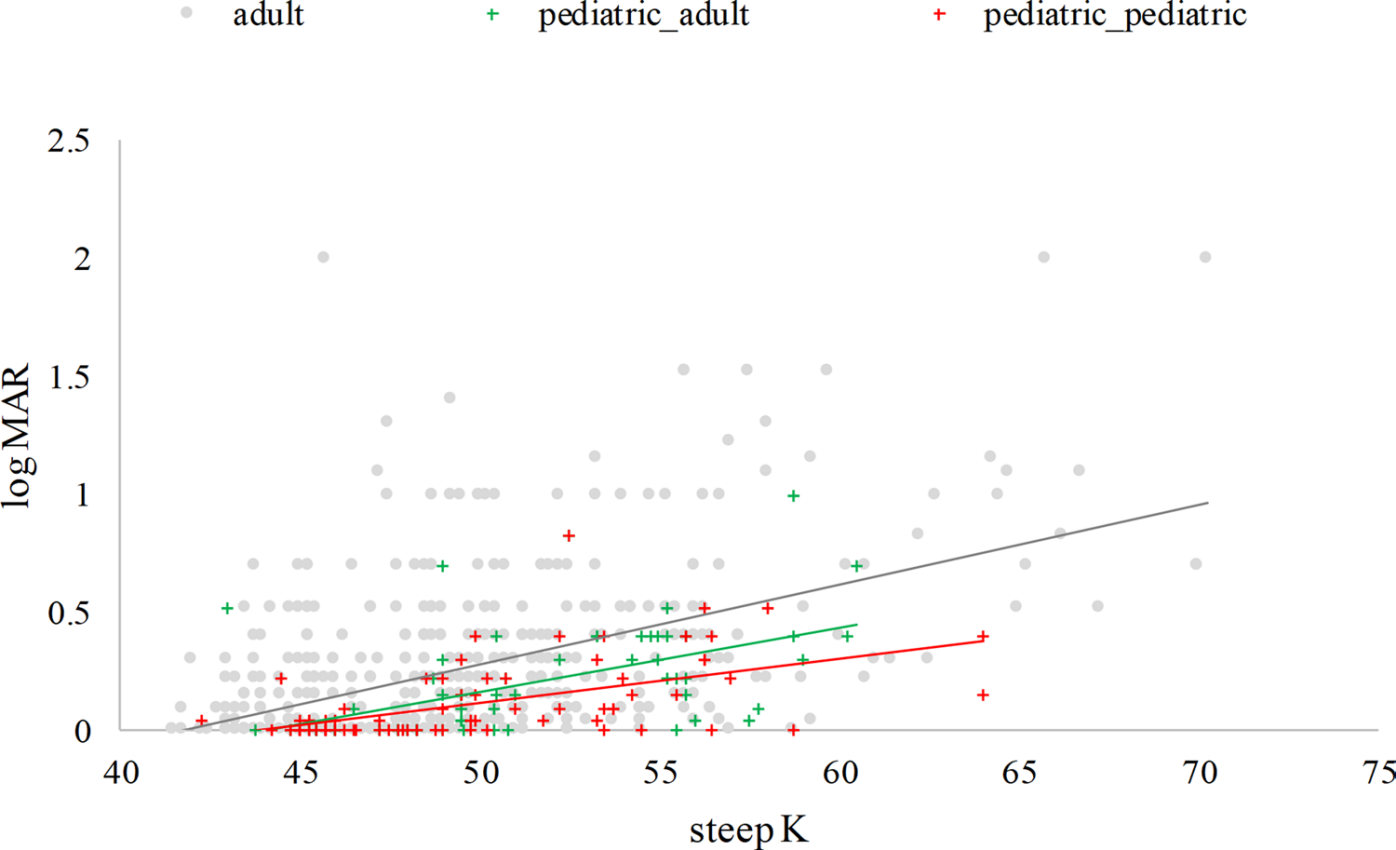
CDVA vs. steep K for the three groups: adult, pediatric_pediatric (the first diagnosis of KC below 18 years old and taking the measurements at age ≤ 18 years old), pediatric_adult (the first diagnosis of KC below 18 years old but taking the measurements at age > 18 years old) (r = 0.48).

Figures 1 and 2 show the CDVA and steep K vs. age. The r between the CDVA and steep K vs. age at measuring were 0.09, 0.14, respectively. It is easily seen that some CDVA and steep K were measured after 18 years old in the pediatric group, and the oldest age was 30 years old. Hence, we further divided the pediatric group into two groups: pediatric_pediatric and pediatric_adult, based on the patient’s age taking the measurements. Figure 3 shows the CDVA vs. steep K for these three groups. The r between the CDVA vs. steep K was 0.48. The GEE analysis reveals a significant difference in the slope between steep K and CDVA among the three study groups. These slopes were 0.0365, 0.0216 (= 0.0365–0.0149) and 0.0122 (= 0.0365–0.0243) for the adult, pediatric_adult and pediatric_pediatric groups, respectively. Three groups presented with progressive steeper K and worsening CDVA with aging and CDVA were negatively correlated with steep K. Considering the visual deterioration, the pediatric-pediatric group showed least affected by steep K compared with pediatric-adult and adult groups. The pediatric-adult group presented with an intermediate effect compared with the other two groups.

$$ \begin{aligned}{\text{log MAR }}  & = -1.5108+0.0365* {\text{steep\_K}} \,(\text{p}<0.0001)+1.0405*{\text{pediatric\_pediatric}}\,(\text{p}=0.0057)\\ &\quad+0.6249* {\text{pediatric\_adult}} \,(\text{p}=0.0310) - 0.0243^*{\text{steep\_K}}\_ {\text{pediatric\_pediatric}}\, (\text{p}=0.0017)\\ &\quad - 0.0149^* {\text{steep\_K}}\_{\text{pediatric\_adult }} (\text{p}=0.0107).\end{aligned} $$

We rewrote this equation for the three groups separately as below:

$${\text{The adult group: log MAR }} = \, - 1.5108 + 0.0365*{\text{ steep}}\_{\rm {K}} \, \left( {{\text{p}} \, < 0.0001} \right)$$

$${\text{The pediatric\_adult group: log MAR }} = \, - 0.8859 + 0.0216*{\text{steep\_K}} \, \left( {{\text{p}} \, = \, 0.0038} \right)$$

$${\text{The pediatric\_pediatric group: log MAR }} = \, - 0.4703 + 0.0122*{\text{ steep\_K}} \, \left( {{\text{p}} \, = \, 0.0082} \right)$$

## Discussion

In this study, we retrospectively analyzed the natural course of KC and compared pediatric and adult patients using data from two tertiary institutions in Taiwan. We found that the clinical characteristics at the initial diagnosis were similar between both groups, but pediatric patients were more likely to exhibit disease progression at the end of follow-up before aggressive treatment.

Although previous studies have been conducted on pediatric KC, they have mainly focused on the outcome of surgical interventions such as CXL, intracorneal ring, and penetrating keratoplasty^24–26^. Our study demonstrated a more rapid progression of sphere and cylinder refractive error, spherical equivalence, steep K, and flat K in pediatric patients with KC than in adult patients from the initial diagnosis to the final visit. Considering that myopia may progress rapidly in patients < 15 years of age^27^, we cannot make any conclusions on the effect of pediatric KC on the change in sphere refractive error or spherical equivalence. There was no significant difference in cylinder refractive error between the two groups at initial diagnosis. However, more cylinder refractive error was noted in the pediatric group than in the adult group at the end of follow-up, which indicates pediatric patients with KC have a more rapid progression of cylinder refractive error than adults. In addition, the changes in steep K and flat K were greater in pediatric than in adult patients. Moreover, our study revealed that the pediatric KC group presented significantly better CDVA at initial diagnosis than the adult group. However, no difference was found at the end of the follow-up period, which indicates that CDVA deteriorated more in the pediatric group. Therefore, the cause of vision deterioration in pediatric KC may be related to cylinder refraction progression and a significant change in steep K or flat K.

Although this has been debated, KC in pediatric patients is more aggressive than in adults. Tuft et al. reported that age < 18 years at the time of diagnosis was an independent risk factor for KC requiring penetrating keratoplasty^28^. However, the studies by Dana^29^ and Lass et al.^30^ did not support this association. A study in Asia showed that KC often progressed rapidly at a younger age and manifested with severe grades^31^. Chatzis and Hafezi reported that the progression of KC was seen in 88% of children in a 1-year study on CXL^32^, while others showed that at the initial diagnosis, pediatric KC tends to be more advanced with more rapid disease progression and profound diminution of vision^33–36^. Our study also reported that pediatric patients had more rapid progression than adults at the end of follow-up.

Little is known about the natural course of KC, particularly in pediatric patients. L’eoni-Mespli’e et al. retrospectively reviewed 49 children with KC aged ≤ 15 years and 167 adults aged ≥ 27 years at diagnosis and found that it was often more advanced in terms of grading slit-lamp biomicroscopic findings and keratometry readings in children^35^. In contrast, there were no differences in the initial diagnosis between the pediatric and adult groups of our study, and pediatric patients had better CDVA and sphere refractive error than adult patients at the initial diagnosis. L’eoni-Mespli’e’s report^35^ found that KC progressed faster in children, with significant differences in the spherical equivalence and maximum and minimum keratometry over a 2-year period, which was in line with our results. Recently, Ferdi et al. conducted a systematic review and meta-analysis on the natural progression of KC and found a significant increase in the *K*_mean_ of 0.4 D at 12 months, a steeper baseline *K*_max_ (> 55 D), and a younger age at presentation (< 17 years) were significantly associated with increased progression (≥ 1.5 D) of *K*_max_ at 12 months^12^. In our study, the pediatric patients had 49.64 D/44.71 D (steep K/flat K) at initial diagnosis, which progressed to 0.74 D/0.60 D at the end of an average follow-up of 3 years. Our pediatric patients seemed to have a relatively benign course compared to the meta-analysis results. This discrepancy may be due to different study designs and populations.

Or et al. compared pediatric KC patients with and without CXL and reported no change in the best-corrected visual acuity (BCVA in logMAR) from baseline to the 5-year follow-up (0.1–0.13)^37^. The Collaborative Longitudinal Evaluation of Keratoconus was a large prospective study of 2418 KC eyes with a mean age of 39.3 years. At the end of the follow-up, the patient’s BCVA lost 2 Snellen letters compared to the baseline, which was quite stable^38^. Wittig-Silva et al. conducted a clinical trial on progressive KC in patients 16 to 50 years of age and showed that at 24 months, the mean cylinder was stable (*p* = 0.15), but there was a significant progression of 1.2 D (*p* = 0.02) at 36 months^39^. Sahin et al. reported the cylinder change from − 4.4 D to − 5.3 D (*p* = 0.04) after a mean follow-up of 24 months using the Orbscan topography^40^. The mean patient age was 32.4 ± 15.2 years, so cylinder changes were presented in pediatric and adult patients. Our study shows no significant changes from the initial diagnosis to final steep K and flat K between pediatric and adult patients, but the progression was more rapid in the pediatric group.

Strictly speaking, there are no clear definitions for KC progression. Based on the Global Consensus on KC and Ectasia Diseases, progression is defined by a consistent change in at least two parameters, which are steepening of the anterior corneal surface, steepening of the posterior corneal surface, thinning, and/or an increase in the rate of corneal thickness change from the periphery to the thinnest point^41^. Several classifications for KC severity have been proposed. The oldest and most commonly used one is the Amsler-Krumeich classification, which includes simulated keratometry and total corneal refractive power^42^. The modified Krumeich classification divides the subjects into four stages depending on clinical characteristics, induced myopia or astigmatism, corneal radii, and thickness^43,44^. Belin and Duncan proposed a new staging system based on anterior and posterior average radii of curvature, thinnest pachymetry, and distance visual acuity^45^. Alio et al. reported a grading system based on the Red Tematica de Investigacion Coopertiva en Slaud classification, a functional scale of corrected VA^46^. Different methods integrating KC’s visual function or topographical characteristics will likely lead to better patient assessment and outcome. This study did not use any score to summarize the KC progressions. For instance, we could not compute the DUCK score because information about the perceived quality of vision was not available in our respective chart data. We could not use the modified Amsler-Krumeich classification either because it is poorly suited for assessing disease progression, and it has limited clinical usefulness for diagnosing earlier stage of KC^22–24^.

In theory, KC is a progressive disease, and thus CDVA is not reversed with aging under natural conditions. In addition, patients with KC have a cylinder refractive error increase, which may lead to vision deterioration^39,40^. In our study, the pediatric group showed less affected CDVA by steep K than adult groups. One of the possible mechanisms is retinal photoreceptor density decreasing with age. Songhomitra reported 55 eyes from human donors aged 18 to 85 years and revealed that the annual photoreceptor cell loss was 0.2–0.4%^47^.

The loss of photoreceptors may be associated with visual functions and necessary for diseases affecting the photoreceptors in elderly patients. Jacob reported the effect of aging and lifestyle on photoreceptors and retinal pigment epithelium over 50 years old and concluded that aging affects photoreceptors^48^. So, the adult KC group may present with worse CDVA than the pediatric KC group under the same steep K (Fig. 3). Age is related to decreasing visual acuity. Ocular aberrations, including astigmatism and higher-order aberrations (HOAs), are associated with deterioration of visual function, and HOA increases with aging^49^. It is also reported that older persons who were free from specific visual pathologies exhibited an age-related decline in presenting far acuity as did those with documented visual pathologies^50^. However, despite the demonstrated loss in acuity with age, the majority of persons maintain at least fair acuity^50^.

The following limitations should be considered when interpreting the findings. First, our study has the inherent disadvantages of retrospective design and restricted clinical data. Many known risk factors of KC, including allergy and asthma, were not examined. In addition, the eye rubbing and atopic dermatitis during the follow-up period were not recorded in the chart. Although we used tomography to confirm the diagnosis of KC, we did not analyze the tomographic data because of the different devices used during the study interval. Instead, we use the steep K and flat K from the autorefractor to measure the corneal curvature from central 3 mm. Hence, the measurement of steep K and flat K may not always reflect the severity of KC. Secondly, the sample size was quite different between the two groups, and the sample size of the pediatric group was relatively small. Thirdly, we did not evaluate the KC variables using fixed time intervals because of the diverse nature of patient visits. The pediatric group had a slightly longer follow-up time than the adult group.

Nevertheless, we believe that the optical variables are still comparable. Fourthly, given that these patients sought treatment at a tertiary center, more severe and progressive cases might be more likely to be referred. Hence, the result of our study might not be generalized to a general population. Furthermore, the socio-economic status might confound our findings. However, it should be minimal because of Taiwan’s universal and mandatory national health insurance since 1995.

In conclusion, our retrospective study demonstrated the clinical presentation of KC in both pediatric and adult patients in Taiwan. There were no significant differences between the groups regarding clinical appearance, comorbidities, cone morphology, or location. Compared to adults, pediatric patients with KC had better CDVA and sphere refractive error at initial diagnosis but progressed more rapidly in refraction through the sphere and cylinder diopter increments, steep K, and flat K deterioration. Since KC is dependent on ethnic differences, our study provides information for ophthalmologists to assess the risk of patients and monitor progression. Early detection, close monitoring, and appropriate interventions preserve vision, particularly in pediatric patients.

## Methods

### Study design and ethical approval

We did a chart review to construct a retrospective cohort study. All the methods described were compliant with the Declaration of Helsinki. Furthermore, the need for informed consent was waived and approved by the Institutional Review Board of Chang Gung Medical Foundation (201601721B0) due to the chart review's retrospective and low harm nature.

### Patient eligibility and keratoconus classification

**Figure 4:**
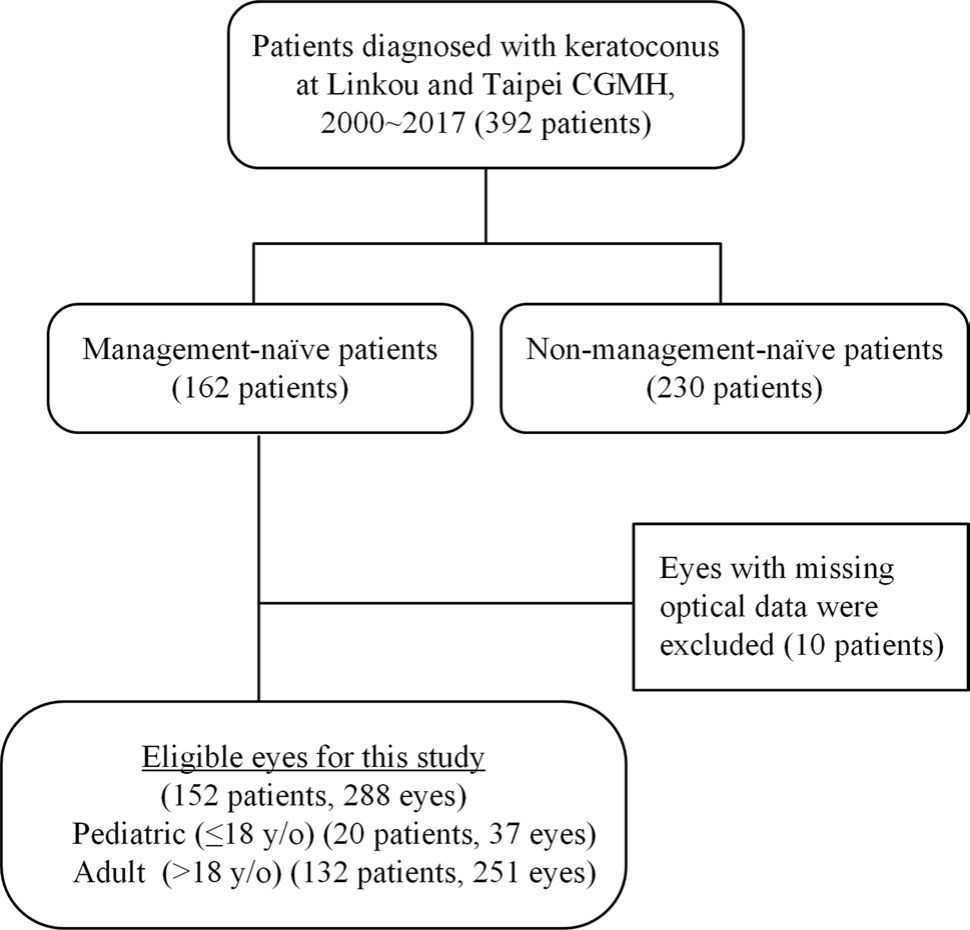
Flowchart of eligible keratoconus patients included in the analysis.

First, we searched the electronic medical records of patients diagnosed with KC (ICD-9-CM: 371.6) during 2000–2017 at the Linkou and Taipei Chang Gung Memorial Hospitals, tertiary care centers in Taiwan. Next, we reviewed the charts to confirm the KC diagnosis by the presence of at least one characteristic slit-lamp biomicroscopic finding, including conical protrusion, Vogt’s striae, Fleischer ring, or any tomographic patterns of KC. The tomographic patterns of KC could be high central corneal power, thin central thickness, or a large difference between the power of the corneal apex and periphery. We excluded patients with a prior history of either diagnosis or treatments for KC at other institutions. Patients were followed from their first visit to our hospital until they were prescribed contact lenses or any surgical treatment. Among 392 patients diagnosed with bilateral KC, 162 were management-naïve patients, and 230 had a treated history from other clinics. After excluding 10 patients with missing optical data, 152 patients (288 eyes) were enrolled in this study. Noted that steep K and flat K information was unavailable in 16 eyes (= 5.3%) (Fig. 4).

We classified the cone shapes as nipple (small and ≤ 5 mm in diameter), oval (large and 5 ~ 6 mm in diameter), and globus (more than 75% of corneal surface affected) according to the classical descriptions of topographic presentations^51^. The tomographic data were derived from three different devices, the combined Placido scanning-slit system (Orbscan II) (Bausch & Lomb, Rochester, NY, USA), the integrated system of Placido disc topography, and dual Scheimpflug tomography (GALILEI G4) (Ziemer Ophthalmic Systems AG, Port, Switzerland), and the rotating Scheimpflug camera (Pentacam) (Oculus, Wetzlar, Germany), in chronological order of device acquisition from 2000 to 2017.

Because all patients in our study received the same steep K and flat K measurement from the autorefractor at the initial and during follow-up visit, we used the steep K and flat K from autorefractor instead of tomography to analysis the corneal curvature in order to reduce inter-machine variation.

### Variables of interest

Demographic and clinical characteristics, as well as optical variables, were reviewed and collected. This included eye rubbing behaviors, comorbidity of atopic dermatitis, date of KC diagnosis, slit-lamp biomicroscopic findings, uncorrected distance visual acuity (UDVA), corrected distance visual acuity with spectacles (CDVA), refraction, keratometry reading (K) including steep K and flat K, and tomographic measurements. The Snellen VA was converted to the minimum angle of resolution VA (Log MAR) logarithm for the statistical analysis.

### Statistical analysis

Descriptive statistics were used, such as mean, standard deviation, and frequency. Scatter plots with correlation coefficient (r) were used to show the strength and direction of the linear relationship between two continuous variables. Wilcoxon rank-sum test or Fisher’s exact test was made to compare the data between the pediatric and adult group, where appropriate. Wilcoxon signed-rank test was made to compare the data within the group, where appropriate. Because of the non-independence of the corrected distance visual acuity (CDVA) of two eyes repeatedly measured over time, we utilized the generalized estimating equation (GEE) to examine the relationship between CDVA and steep K. The within-subject correlation matrices in GEE were exchangeable^52^. This significance level (or type I error) was set at 0.05. We used a sequential Bonferroni-type procedure for multiple testings to maintain the overall type I error of 0.05. The p values of multiple tests in Tables 1 and 2were ranked from smallest to largest as p(i), where i is the rank. Only p values < i/(46 = number of multiple tests)* 0.05 were considered statistical significance. Hence, the borderline significance was set between 0.05 and *p* values < i/46. All statistical analyses were performed using the SAS version 9.4 (SAS Institute, Cary, NC, USA).

## Data Availability

The data that support the findings of this study are available on request from the corresponding author.
